# Bottom-Up Fabrication of DNA-Templated Electronic Nanomaterials and Their Characterization

**DOI:** 10.3390/nano11071655

**Published:** 2021-06-23

**Authors:** Chao Pang, Basu R. Aryal, Dulashani R. Ranasinghe, Tyler R. Westover, Asami E. F. Ehlert, John N. Harb, Robert C. Davis, Adam T. Woolley

**Affiliations:** 1 Department of Chemistry and Biochemistry, Brigham Young University, Provo, UT 84602, USA; mpangchao@gmail.com (C.P.); aryalbasu99@gmail.com (B.R.A.); dulashani13@gmail.com (D.R.R.); asami.ford@gmail.com (A.E.F.E.); 2 Department of Physics and Astronomy, Brigham Young University, Provo, UT 84602, USA; tyler.westover13@gmail.com (T.R.W.); davis@byu.edu (R.C.D.); 3 Department of Chemical Engineering, Brigham Young University, Provo, UT 84602, USA; john_harb@byu.edu

**Keywords:** DNA origami, nanofabrication, DNA templates, electrical characterization

## Abstract

Bottom-up fabrication using DNA is a promising approach for the creation of nanoarchitectures. Accordingly, nanomaterials with specific electronic, photonic, or other functions are precisely and programmably positioned on DNA nanostructures from a disordered collection of smaller parts. These self-assembled structures offer significant potential in many domains such as sensing, drug delivery, and electronic device manufacturing. This review describes recent progress in organizing nanoscale morphologies of metals, semiconductors, and carbon nanotubes using DNA templates. We describe common substrates, DNA templates, seeding, plating, nanomaterial placement, and methods for structural and electrical characterization. Finally, our outlook for DNA-enabled bottom-up nanofabrication of materials is presented.

## 1. Introduction

Conventional top-down fabrication of nanoscale electronic structures involves highly sophisticated methods and requires an extremely clean, low-particulate working environment. In addition, top-down fabrication is based on photolithography, which utilizes toxic chemicals that raise costs related to environmental issues [1]. Importantly, promising efforts in bottom-up nanofabrication have emerged in the past two decades. Among these alternative strategies, DNA-based nanofabrication for synthesis of engineered nanostructures offers many advantages such as a high degree of design flexibility, self-assembly, and low-cost manufacturing [2,3,4]. This versatile DNA-based approach arranges materials by mimicking Nature’s principle of self-assembling organized structures from a disordered collection of smaller parts. Metals, semiconductors or other materials can self-assemble on DNA templates, and these nanoscale materials can be grown to form continuous nanostructures under chemical treatment [5].

DNA molecules have been used as constituents for synthesis of frameworks since Seeman’s initial work in 1982 [2]. Both double-stranded DNA (dsDNA) [6] and single-stranded DNA (ssDNA) [7,8] have been used as building blocks for self-assembling nanostructured templates. DNA templates offer great potential to control the arrangement of electronic nanomaterials such as metals and semiconductors [8]. These properties provided a basis for promising initial nanowire (NW) synthesis: DNA-templated silver [9] and gold NWs [10], which then were followed by the demonstration of DNA-templated carbon nanotube (CNT) field-effect transistors [11]. Subsequently, palladium [12], copper [13], platinum [14], nickel [15], and cobalt [16] NWs were likewise selectively created on DNA. 

A revolutionary technology that accelerated the large-scale research of DNA nanofabrication was shown by Rothemund [17] in 2006 and named “DNA origami”. With this method, nanoscale shapes and patterns of two-dimensional (2D) DNA templates 100 nm in size were precisely designed and controlled with 6 nm spatial resolution through complementary hybridization between a long ssDNA and many short synthetic ssDNAs. On the basis of this, Li et al. [18] built a large 2D array by assembling several DNA origami nanostructures together via the connection of staple strands. However, completing the complex design was a tedious and error-prone task without aid from a computer. Douglas et al. [19] developed an open-source program named caDNAno to significantly reduce the workload during designing and help expand into three-dimensional (3D) applications of DNA origami [20]. DNA origami has advantages in precise positioning and organization of electronic materials with different morphologies. Therefore, selective metallization of DNA origami templates is desirable [21,22,23,24]. Pilo-Pais et al. [25] created precise binding sites on a DNA origami template by extending the staple strands with a predesigned sequence pattern. On the basis of these techniques, various electronic materials have been controllably attached to DNA origami. In contrast to previous duplex DNA-based template techniques, DNA origami can also be used to pattern inorganic materials [26,27] and CNTs [28]. However, metallization of DNA origami nanostructures could be more broadly used in assembling nanoelectronic circuits [29] and plasmonic structures [30].

**Figure 1:**
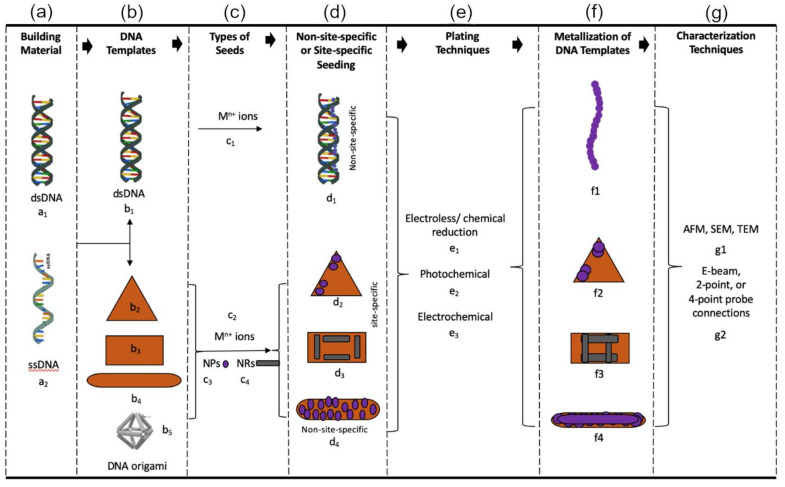
Schematic of DNA-templated material assembly. (**a**) DNA strands serve as a building material using (a_1_) dsDNA or (a_2_) ssDNA. (**b**) The DNA strands form templates: (b_1_) a dsDNA or (b_2–5_) DNA origami structures: triangle, rectangle, bar, or octahedron. (**c**) Types of seeds: (c_1_,c_2_) metal cations in solution, (c_3_) inorganic nanoparticles, or (c_4_) inorganic nanorods. (**d**) Seeding methods: (d_1_) non-site-specific seeding on a dsDNA and (d_2–4_) site-specific seeding on DNA origami: triangle, rectangle, or bar. (**e**) Plating techniques: (e_1_) electroless/chemical reduction, (e_2_) photochemical, and (e_3_) electrochemical. (**f**) Metallized DNA templates: (f_1_) dsDNA, (f_2–4_) DNA origami structures: triangle, rectangle, or bar. (**g**) Characterization techniques: (g_1_) AFM, SEM, or TEM, or (g_2_) electron-beam formation of 2-terminal and 4-terminal probe connections.

The process of DNA-templated construction includes seven steps from initial material treatment to final nanostructure characterization. An overview of the process of making DNA-templated nanomaterials is shown in Figure 1. In the first step, DNA (either single- or double-stranded) is used as the fundamental building material. M13mp18 is a widely used ssDNA in DNA origami; lambda (λ) DNA is a common linear dsDNA that can be implemented for nanomaterial assembly. The second step is template fabrication, which can be achieved by DNA origami or duplex DNA. During the third step, a seeding solution is prepared, which contains metals or other compounds such as ions, nanoparticles, or nanorods. The fourth step is to deposit these seeds onto the template via non-site-specific or site-specific methods, followed by the fifth step, plating. Various techniques such as electro/electroless or photochemical plating can be applied in this process. These steps provide a process to place materials onto a DNA nanoarchitecture that can be further characterized through atomic force microscopy (AFM), scanning electron microscopy (SEM), transmission electron microscopy (TEM), or electrical measurement.

In this review, we describe recent progress in DNA-templated fabrication of electronic nanomaterials and their characterization. The paper is divided into two principal sections: Fabrication and Characterization. In the Fabrication section, we describe substrates, DNA templates, and metallization or placement of metals and other conductors on novel DNA nanostructures. Among electronic materials, we focus on metals, semiconductors, and CNTs; and summarize achievements in the fabrication process. We cover structural approaches in the Characterization section, including AFM, SEM, and TEM. We also discuss electron beam lithography and electron beam induced deposition in their role of facilitating electrical characterization. Finally, we offer our outlook for DNA-based nanofabrication. The overall goal of the paper is to help the reader better comprehend DNA-templated materials nanofabrication and characterization by seeing its history, progress, and future prospects.

## 2. Fabrication

### 2.1. Substrates

DNA serves as a useful template for creating nanodevices and structures because of its programmability and structural stability. The stability of DNA when bound to a substrate makes both the substrate and the DNA nanostructure important topics of study. 

A key challenge with DNA deposition is determining the conditions required to anchor the DNA strongly onto the substrate, with the proper orientation. Pillers and Lieberman [31] found that DNA on mica retained its chemical and physical stability under temperature conditions as high as 150 °C for 45 min. They further generated silicon carbide by annealing DNA origami at high temperatures on silicon. To accomplish this, rectangular DNA origami structures were deposited onto 1 cm^2^ cleaned silicon chips, followed by the deposition of 50 nm of SiO_2_. Heating the samples at 900–1100 °C enabled carbon atoms to diffuse from the DNA origami into the Si substrate to produce SiC structures that retained the shape of the DNA origami. The resulting process represents a novel technique for nanoscale patterning of SiC.

DNA surface deposition can be affected by a variety of factors, including the preparation of the DNA solution and the substrate itself, along with the deposition technique. Mica and silicon are commonly used substrates. When depositing DNA templates onto a substrate, Mg^2+^ is used to give the substrate a positive charge. The phosphate backbone (negative charge) on the DNA can then bind electrostatically to the positively charged substrate. Gopinath et al. [32] varied the Mg^2+^ concentration and binding site length to determine optimal conditions for triangular DNA origami to be placed onto Si/SiO_2_. They were able to control the binding site of DNA using Mg^2+^ concentrations as low as 35 mM. Linko et al. [33] used a spray-coating technique to deposit DNA onto untreated silicon and glass surfaces. The solution sprayed on glass required a concentration 10 times greater than that sprayed onto silicon, but both yielded surface coverage of about 4 objects/µm^2^ due to the homogenous layering of the DNA origami nanostructures. The placement achieved by this approach produced a single-layered sample. This method can be easily scaled up, may be more cost-effective than previous techniques, and can be utilized on a variety of substrates, including silicon, glass, and plastics. In these ways, DNA can be stabilized on a variety of substrates, allowing use in selectively placing nanomaterials.

### 2.2. DNA Templates

DNA is an ideal nanomaterial that can be used in bottom-up nanofabrication to create self-assembled 2D or 3D architectures by hybridization base pairing. DNA nanostructures enable programmed positioning of nanoscale features with high spatial resolution, which is promising for constructs such as nanowires, nanodevices, plasmonics, and sensors. DNA templates can be formed in multiple ways. Katrivas et al. [34] described a PCR method for synthesizing dsDNA using short strands of 6–15 base pairs in repeat to form linear dsDNA that is thousands of base pairs long. Oligonucleotides with a half-complementary 5′-end can be formed into long dsDNA via hybridization, and then be enzymatically converted into non-nicked dsDNA. This DNA can form templates for wires or transistors, which are used in nanoelectronic devices and circuits. Brun et al. [35] formed metal NWs using λ-DNA bundles. The DNA solution was dropped on electrodes and allowed to evaporate, leaving a template; during deposition the metal self-aligned with the DNA template. This approach successfully formed small-scale NWs 10–100 nm in length. Gür et al. [36] also utilized DNA bundles, depositing gold nanoparticles (NPs) onto tubular six-helix DNA bundles with eight binding sites, resulting in a yield of 98.7%. This high yield is promising for the production of more reliable multiparticle plasmonic devices. 

#### 2.2.1. DNA Origami

DNA origami refers to folding of a long ssDNA into desired structures in the presence of a specific set of shorter DNA strands. Rothemund [17] first demonstrated the DNA origami technique for folding and creating nearly arbitrary 2D configurations by exploiting the self-assembly properties of DNA. Since then, DNA origami has generated both 2D and 3D DNA nanostructures with a wide range of applications. For example, innovative DNA origami designs have been applied to create templates that can be converted into functional conductive nanostructures. Mathur et al. [37] created triangle and rhombus DNA origami templates containing protruding base sequences at binding sites for metal attachment. The outer edge length of their equilateral triangle was 120 nm with an internal triangular hole approximately 60 nm long. Their DNA rhombus was generated by using bridging staples in the DNA triangles. This team attached DNA functionalized gold nanorods (NRs) and gold NPs to the origami to generate different DNA-templated plasmonic nanostructures. Shen et al. [38]. utilized a single-layer rectangle (92 nm × 72 nm) and cross-shaped, double-layered tiles (95 nm × 30 nm) to create DNA origami silhouettes with gold, copper, and silver deposition. This technique exploits DNA origami shapes to create uniform metallic nanostructures on a silicon surface. 

3D DNA origami templates are increasingly being employed in association with other nanomaterials. Schreiber et al. [39] demonstrated a DNA origami flower structure having a DNA-coated Au NP at the middle point. Approximately 20 nm long bundles of four DNA helices present in a single DNA origami were used as radial petals that wrapped the Au NPs via complementary DNA hybridization to form a composite flower shape.

#### 2.2.2. DNA Origami Stability

DNA origami need to be sturdy enough to maintain their initial shape during the metallization process; factors that can change the shape of DNA origami include solution concentrations, temperature, and seed morphology. In addition, DNA origami templates are assembled with a long ssDNA that is folded and held together by many shorter staple strands; the structure may unfold under some conditions. Kim et al. [40] systematically analyzed possible physical and chemical treatments that could degrade the structural stability of DNA origami, which include temperature, pH, or exposure to organic solvents or UV/O_3_. They found that shapes of DNA nanostructures were intact under conditions up to 200 °C for 10 min, 24 h exposure to alkaline, and 5 min exposure to UV/O_3_. Kim et al. [41] also developed an approach to increase the stability of DNA nanostructures toward chemical or mechanical stresses. They deposited an Al_2_O_3_ atomic layer on top of DNA templates, which acted as a protective coating against UV/O_3_ oxidation. This film also increased the stability of both DNA nanotubes and DNA origami when exposed to UV/O_3._ The only downside of this approach was that some parts of the nanostructures were damaged during lift-off from the polymer substrates.

Of all the conditions that damage DNA origami templates, elevated temperature is the most common and problematic one because the typical melting temperature of staple strands in DNA is <80 °C [42]. Generally, DNA origami in solution starts breaking apart at about 50 °C and will completely denature at 70 °C [43]. However, during DNA origami assembly, annealing is done in the presence of excess staple strands, making it useful for growing well-formed nanostructures [44,45,46]. Pillers and Lieberman [31] studied thermal stability of DNA origami on the most common substrate, mica, using AFM and X-ray photoelectron spectroscopy to detect structural and chemical changes. Their results showed that common DNA origami templates remained stable at temperatures of up to 150 °C used during general lithographic processing.

Besides the effects of elevated temperatures, scientists also analyzed the influence of low temperatures on DNA origami, which are important for long-term storage in various technological or medical applications. Xin et al. [47] studied the effects of repeated freezing and thawing cycles on both 2D and 3D DNA origami nanostructures. They found that 2D and 3D DNA origami can tolerate at least 32 freeze–thaw cycles without changing structural integrity. Almost 85% of the 3D nanostructures remained intact even after 1000 freeze–thaw cycles with the addition of the cryoprotectants, glycerol and trehalose. These results show that cryopreservation could be an effective way to facilitate DNA origami long-term storage.

Salt concentration is another factor that may affect the structural stability of DNA origami. High Mg^2+^ concentrations are typically used for assembling DNA origami. However, Kielar et al. [48] demonstrated that DNA origami templates can also remain stable even in Mg^2+^ concentrations as low as 10 µM when mixed with 10 mM Tris buffer at pH 8.9; some templates maintained their shapes for as long as two months. Three different shapes of DNA origami (triangle, and 6- or 24-helix bundles) were tested in different buffer systems with concentrations of Mg^2+^ as low as 10 µM and high concentrations of monovalent cations. The results show that high Mg^2+^ concentrations are not necessary to maintain the stability of DNA origami. Kielar et al. [49] also used AFM to investigate the effects of staple strand age on the stability of DNA origami structures. They found that cryopreservation storage slowly degraded the staple strands, which caused the assembled DNA origami to become less stable. Their results further showed that staple-related effects causing damage to DNA origami templates depended on the superstructures themselves.

#### 2.2.3. DNA Nanotubes

DNA nanotubes are another class of DNA template for fabricating useful structures. DNA nanotubes can be formed by using single-stranded or multi-crossover tiles, DNA origami, or multiple rungs. Teschome et al. [50] demonstrated Au NP alignment on DNA origami nanotubes fabricated with six-helix bundles. More rigid DNA origami nanotube structures would be beneficial in minimizing deformation of the completed structures in future applications. Particularly, applications that depend on arrays of parallel NP chains or NWs over large surface areas, such as transparent conductive electrodes, may find this technique to be useful. Gür et al. [36] reported a method for self-assembling plasmonic waveguides on DNA origami nanotubes. They also used six parallel interconnected helical bundles of DNA origami as the templates. The deposition of Au NP decorated DNA nanotubes on substrates still needs better surface placement and alignment, as they created aggregated structures on the surface. Recently, Ranasinghe et al. [51] formed conductive NW structures on DNA nanotubes using Au NRs and Pd seeds, combined with Au electroless plating. These DNA nanotubes were designed to connect in an end-to-end fashion, providing bridges with different separation distances and relative orientation [52]. 

Our summary of the details about DNA templates and substrates is tabulated in Table 1, which includes dimensions and shapes for the three main types of DNA templates, along with details about the attached materials. In next section, we focus on various conductive nanomaterials linked to DNA templates, as well as seeding techniques and plating methods.

## 3. Metallization of DNA Templates

DNA templates can accommodate nanomaterials in various forms, such as ions, NPs, and NRs. As such, they can help to create conductive structures through seeding and metallization approaches. In many cases, DNA templates dispersed in liquid are deposited onto substrates by exploiting electrostatic interactions between the negatively charged DNA backbone and a positively charged substrate such as Si or mica after treatment with divalent cations. Seeding, which is the process of binding nanomaterials to DNA templates, is performed by depositing seeding materials through non-site-specific or site-specific interactions with DNA (Figure 1c,d). DNA templates can either be incubated with a seeding solution, or the seeding solution can be added to a substrate with pre-deposited DNA. Seeding is a critical process because it guides the fabrication of conductive structures in subsequent steps. Plating fills gaps and connects nanomaterials by either isotropic or anisotropic metal deposition (Figure 1e,f). Continuous and conductive structures are made by eliminating gaps between nanomaterial seeds on the DNA templates. 

### 3.1. Alignment

**Figure 2:**
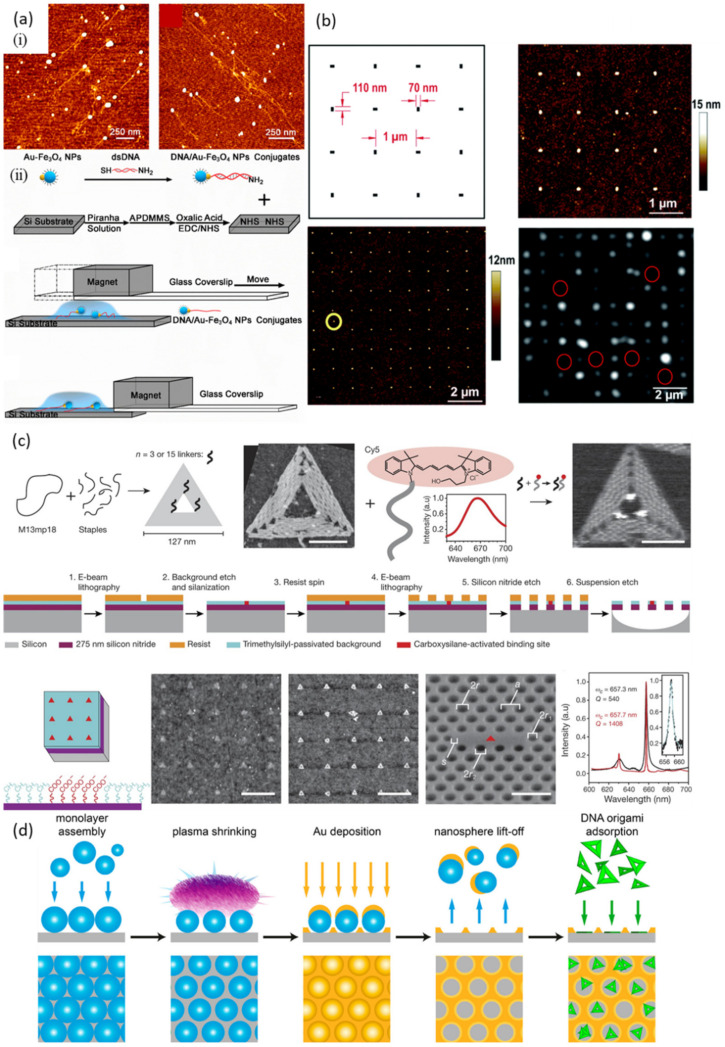
DNA alignment techniques. (**a**) (i) AFM images of dsDNA/Au−Fe_3_O_4_ NP conjugates aligned on silicon substrates under magnetic fields in different directions. (ii) Immobilization procedure for dsDNA/Au−Fe_3_O_4_ NP conjugates on silicon substrates. Reprinted from Liu et al. [71] Copyright 2018 American Chemical Society. (**b**) Directed assembly of porphyrin-labeled DNA origami on a Teflon resist after e-beam exposure. Reprinted from Shaali et al. [72] Copyright 2017 Royal Society of Chemistry. (**c**) DNA origami with single-stranded linkers and attached Cy5 placement on binding sites created via e-beam patterning to make negatively charged carboxylate groups in a background of hydrophobic methyl groups. Reprinted from Gopinath et al. [32] Copyright 2016 Springer-Nature. (**d**) Experimental strategy for making a hexagonally ordered pattern of nanoholes in a thin Au film on a Si wafer, which is used to direct the adsorption of DNA origami nanostructures. Reprinted from Brassat et al. [69] Copyright 2018 American Chemical Society.

A major issue in bottom up nanofabrication is the precise alignment or placement of DNA; oftentimes, elongated DNA is more useful than coiled DNA on surfaces. Liu et al. [71] aligned long DNA chains reversibly by tethering Au/Fe_3_O_4_ magnetic NPs (Figure 2a) to DNA via Au-S bonds, with the other ends attached to the silicon substrate via amide bonds. The DNA bound to silicon could be moved under an applied magnetic field but restored back to the coiled state upon removal of the magnetic field. This method was useful for studying DNA interactions with drugs and proteins using AFM. The combination of bottom-up self-assembly and top-down lithography for providing precise locations for DNA on a surface is also a useful strategy. Shaali et al. [72] performed deposition of DNA origami modified by hydrophobic anchors with surface coverage of ~80% of sites in a Teflon negative electron beam (e-beam) resist pattern. Figure 2b shows a schematic of the nanopattern designed to localize DNA structures and an AFM image of the surface after e-beam exposure and development of a 35 nm Teflon resist. Moreover, Shetty et al. [73] used nanosphere lithography to place DNA origami on surfaces. They used AFM and SEM to characterize binding site sizes with different nanosphere diameters. The methods may facilitate the placement of 2D and 3D DNA nanostructures for single-molecule techniques. Gopinath et al. [32] placed DNA origami onto target sites created by e-beam patterning as shown in Figure 2c. This approach has potential for use in rapidly prototyping hybrid nanophotonic devices. Brassat et al. [69] studied the adsorption of DNA origami triangles in nanoholes fabricated by nanosphere lithography in thin Au films on Si wafers as shown in Figure 2d. They studied the impact of buffer conditions on DNA origami adsorption in the nanohole arrays and observed partial adsorption of one or more DNA origami triangles inside the nanoholes with a part of the triangle crossing the boundary to the Au film, even under optimized conditions. Partial DNA origami adsorption might be avoided by using multi-layer DNA origami rather than single-layer DNA origami.

Block co-polymers (BCPs) have gained attention in thin-film nanolithography because they spontaneously form highly uniform nanofeatures at the resolution scale of current lithographic tools. Pearson et al. [74] showed directed assembly of DNA origami on chemically functionalized, BCP-generated Au NPs. The results indicated stable, highly selective attachment to the surface through the base pairing of DNA origami rectangles with patterned ssDNA on the surface. Further control of DNA origami placement might be achieved by using multiple complementary regions with different base sequences. Zhang et al. [75] used cholesterol-DNA with specific sequences to form spherical micelles and nanorods in aqueous solution. They adjusted the design of the structures through pH, DNA sequence, length of the connecting spacers and the salt concentration. They studied the hierarchical assembly that occurs between DNA strands and cholesterol hydrophobic cores. This is an encouraging step toward building different types of nanostructures with potential for use in drug delivery applications.

### 3.2. Seeding

#### 3.2.1. Ionic Seeding

Ionic seeding was employed in the first publication on DNA-templated Ag nanowires [9], a key advancement that led to subsequent efforts to construct nanowires and heterostructures on DNA. In ionic seeding, metal ions interact electrostatically with the phosphate backbone of DNA. Different procedures have been utilized to incorporate metal ions, such as silver, into DNA strands to construct metal nanostructures. Kasyanenko et al. [76] bound bis(1,10-phenanthroline) silver(I) (Ag-Phen) to DNA in solution where the phenanthroline ligands allowed for uniform binding of silver on DNA structures. The Ag-Phen-DNA complexes were treated with sodium borohydride to reduce Ag metal on DNA fibrils. Vecchioni et al. [77] reported intercalation of Ag^+^ into cytosine-mismatched DNA duplexes, which after silver plating, demonstrated a significant enhancement in single-molecule conductivity. González-Olvera et al. [78] showed binding of metal cations such as Ag^+^, Zn^2+^, Fe^2+^, and Cu^2+^ with deprotonated guanine or thymine groups on short ssDNA. They synthesized silver, zinc, and copper NPs by reducing their cationic aggregates in alkaline aqueous solution. This technique of DNA metallization might find use in the DNA-assisted fabrication of nanostructures. Kasyanenko et al. [79] demonstrated the formation of complexes with nitrogen bases on DNA by using Ag^+^ and Au^3+^, and reduced these ions into Ag NPs (2–10 nm) and Au NPs (3–6 nm), but the NPs were not constructed into NWs. Ganguly et al. [53] incorporated borane phosphate DNA onto CNTs; when Ag^+^, Au^3+^, or Pd^2+^ was incubated with the constructs they were reduced into NPs because of the incorporated reducing equivalents. Almaky et al. [80] utilized N-(3-pyrrol-1-yl-propyl)-2,2′-bipyridinium hexafluorophosphate to link Cu^2+^ and Pd^2+^ with DNA through 2,2′-bypyridine metal-binding sites. A reducing agent converted the metal ions into NWs through nucleation and growth processes. Electrical characterization revealed that the conductivity of a Cu NW was 1.6 ± 0.3 S cm^−1^, whereas that of a Pd NW was inconsistent. Dugasani et al. [81] demonstrated fabrication of Cu^2+^-DNA hybrid fibers in large scale. Cetyltrimethylammonium chloride-associated DNA was formed, in which Cu^2+^ was bound to phosphate backbone groups. Structural characteristics of Cu^2+^-coated DNA were determined, but the Cu^2+^ was not reduced to copper metal. Yoo et al. [82] constructed Co^2+^, Cu^2^, Tb^3+^, and Eu^3+^ co-doped DNA nanostructures. They collected current-voltage (I–V) curves from the DNA-templated structures and studied their topological and optical properties. 

Both chemical and electrochemical reduction methods have been utilized to form DNA-templated nanostructures. Mohamed et al. [83] bound Rh^3+^ to λ-DNA, followed by chemical reduction in the presence of NaBH_4_ in solution to generate Rh NWs. They also used an electrochemical reduction method in which they deposited a DNA/Rh^3+^ reaction solution on an n-type Si wafer which acted as the working electrode to reduce Rh^3+^ onto DNA. Samples from both chemical and electrochemical reduction were electrically characterized with scanning conductance microscopy and conductive AFM. Yamada et al. [84] chelated Hg(II) ions between TT sequences of DNA in solution. They measured the electrical conductance of the Hg-DNA complex in buffer solution by using break junction STM, which was effective in characterizing electronic behavior in metal–DNA complexes.

Despite difficulties associated with location-specific binding of precursor ions, several articles have been published on the metallization of DNA origami by ionic seeding. Ouyang et al. [85] utilized 16-nm-wide DNA nanoribbons with non-templated binding sites for Cu^2+^, which were reduced to Cu NPs and formed nanoclusters. The DNA-templated Cu structures had enhanced luminescence and florescence stability, although their electrical behavior was not studied. Jia et al. [86] demonstrated localized binding of Cu^2+^ and Ag^+^ with protruding DNA sequences on 2D DNA origami via N–ion interactions. Subsequent metallization was performed through reduction-generated site-specific metal patterns with ~10 nm resolution. Electrical characterization of the structures was not performed, but this strategy could be used to fabricate nanoscale metal features for future nanoelectronics. 

#### 3.2.2. Site-Specific Seeding

Site-specific seeding is used for making complex conductive structures because of precise control of material positioning. Different species such as metal NPs [25,66,87,88,89] or NRs [56,60,90,91] can be used as seeds to attach on DNA at specific locations. A common strategy for site-specific attachment of Au seeds to a DNA origami template is to use thiol-modified DNA to form Au NP-DNA conjugates [25]. Gates et al. [65] investigated parameters such as time of hybridization, ratio of seeds to templates, and concentration of Mg^2+^ to optimize the seeding density and yield. They found that 30–90 min hybridization times, 1.8–4.5 ratio of Au NPs per attachment location, and 70–100 mM Mg^2+^ were best suited for making thinner Au NWs that appeared continuous in SEM imaging. 

**Figure 3:**
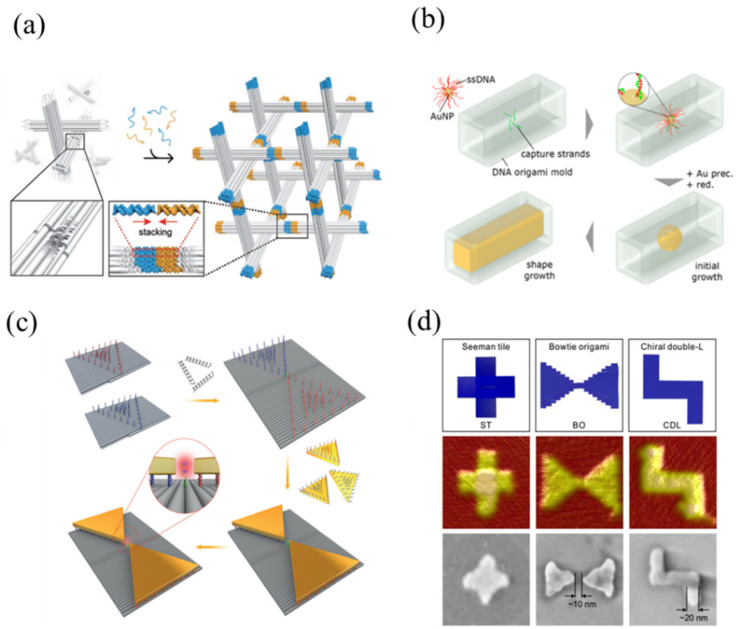
Site-specific seeding on DNA origami templates. (**a**) Schematic illustration of triangular DNA origami building block design and assembly. Reprinted from Zhang et al. [61] Copyright 2018 Wiley-VCH Verlag. (**b**) Mold-constraint growth procedure for gold nanostructures. Reprinted from Helmi et al. [92] Copyright 2014 American Chemical Society. (**c**) Gold bowtie nanostructures based on DNA origami: representation of the experiment. Reprinted from Zhan et al. [62] Copyright 2018 Wiley-VCH Verlag. (**d**) DNA origami designs and a step-by-step fabrication procedure for different nanostructures. Reprinted from Shen et al. [59] Copyright 2018; distributed under a Creative Commons Attribution License 4.0 (CC BY).

Beyond 2D structures, site-specific seeding can also be used to create 3D arrangements. Zhang et al. [61] utilized triangle-shaped DNA origami to arrange Au NPs in predesigned 3D rhombohedral crystalline lattices (Figure 3a). This route for 3D lattice building can produce large-scale structures with precise control, potentially allowing the formation of single crystals hosting different metal, non-metal, or semiconductor components. The size of the DNA origami monomer is restricted by the length of the scaffold strand, limiting hosting spaces, so for future practical applications λ-phage scaffolds with hierarchical assembly could be advantageous.

A 3D DNA origami nanostructure with an internal cavity can serve as a mold to first hold metal NPs and then let them electrochemically grow to form NRs whose size and shape are limited by the cavity (Figure 3b) [92]. Au NP seeds with a complementary sequence were hybridized with capture DNA strands at predesigned binding sites within the DNA origami mold. Bayrak et al. [68] utilized a similar approach but coupled DNA origami mold monomers into a longer linear structure to assemble conductive, micrometer-long Au NWs. These NWs were formed using 5 nm DNA-functionalized Au NPs which have fewer interfaces for improved conductivity. However, the resistance at the mold–mold interfaces was still high, and yields for long DNA mold templates need to be improved. Ye et al. [93] improved the assembly yield for five-mold-long DNA structures up to 79% by establishing four unique, orthogonal, high-affinity interfaces among the individual mold elements, which are aligned and precisely assembled. This programmable pattern assembly allowed NW length control. Sun et al. [94] assembled a nano-mold with a 3D cavity that was used for synthesis of distinct silver nanostructures, such as equilateral triangles, cuboids, and spheres. These structures held either Ag NPs or Au NPs as seeds, and the seeds grew into 3D composite structures that exhibited plasmonic properties. 

Zhan et al. [63] utilized Au NRs and DNA origami tripods to construct three 3D plasmonic nanostructures with different inter-NR angles by controlling their relative orientations and distances. Configurations displayed different light scatting spectra with strong resonance peak splitting, in good agreement with calculations. These plasmonic nanostructures have potential for optical signal transmission applications in single-molecule fluorescence detection. In addition, Zhan et al. [62] successfully attached gold nano-prisms on binding sites on bowtie DNA origami and precisely anchored a single Raman probe at the gap between the two triangles (Figure 3c). These metallized gold bowtie nanostructures acted as nanoantennae to amplify single-molecule surface-enhanced Raman scattering by a factor of 10^9^. This method could be used to optically monitor chemical reactions. Shen et al. [59] harnessed the unique optical properties of Au-metallized bowtie-shaped DNA origami nanostructures, combined with conventional lithography to create nanoantennae with a chiral plasmonic response for surface-enhanced Raman spectroscopy (Figure 3d). Piskunen et al. [57] fabricated bowtie-shaped antennae on silicon nitride and sapphire substrates, for use as molecular sensors. The patterning shapes of these metal nanostructures can be precisely controlled through the DNA origami design, which is used to create the thin film SiO_2_ pattern in which the metal structures are formed, offering a general tool for high-throughput nanopatterning. Adaptation of more standard SiO_2_ thin film growth methods to work with this process would further facilitate its use. Gür et al. [36] systematically investigated factors that can affect the yield for site-specific arrangement of Au NPs on DNA origami to understand defect formation. They studied solution ionic strength, stoichiometric ratios, assembly kinetics, and oligonucleotide linkage to optimize the protocol for assembling plasmonic devices. Au NPs with up to 56 nm diameters were assembled into structures with yields up to 98.7%; future efforts should focus on the application of these protocols to smaller-diameter Au NPs.

An ongoing challenge with DNA-based nanofabrication is the creation of long NWs of uniform thickness and good electrical connectivity. Shen et al. [95] designed a hexagon tile shaped DNA nanostructure, which hierarchically self-assembled into nanometer-sized honeycomb array clusters and 2D micrometer-sized tube lattices using connector strands between tile monomers. DNA-functionalized Au NPs were directly attached to sequence-specific DNA hybridization sites designed on the hexagon tiles. Although many Au NPs were specifically bound to the templates, there were gaps between seeds, and no continuous wires were formed; thus, this method may create larger architectures, but sensor applications would be likely limited by gaps between Au NPs.

Site-specific seeding can also be used for attaching metal oxides, semiconductors, polymers, or CNTs. Li et al. [58] utilized the hybridization between thiol-containing ssDNA and protruding staple sequences on DNA origami to create seeding sites for synthesis of Pd and Fe_2_O_3_ nanoclusters. In addition, they fabricated “I”, “L”, and “U” structures on DNA tiles, and assembled nanoclusters on them. This method could potentially be applied to the synthesis of other metal or semiconductor nanoclusters that have strong affinity for thiol groups. Zessin et al. [96] synthesized thiophene-based polymers with an attached DNA sequence and seeded these block copolymers on specific sites on DNA origami. The optical properties of the conjugated polymers were influenced by π-π stacking, which yielded fluorescence signal enhancement. This result has potential to extend DNA-templated bottom-up assembly to DNA−polymer hybrid structures, for future synthesis of nanophotonics with switchable functions. 

## 4. Plating

### 4.1. Electroless/Chemical Reduction

Electroless plating is widely used in bottom-up NW fabrication due to its ability to form thin layers of uniform thickness and to deposit material onto complex templates with nanometer features [97]. Electroless plating, also known as chemical plating, involves a redox reaction induced by a chemical reducing agent that provides electrons to reduce metal cations in solution to form metallic deposits (see Equation (1)). In this autocatalytic process, the metal acts as a catalyst to further drive metal cation reduction to accumulate more metal on the surface.

(1) $$Metal\;Ion\;\left(aq\right)+Reducing\;Agent\;\left(aq\right)\to \;Metal\;\left(s\right)+Oxidized\;Product\;\left(aq\right)$$

Pearson et al. [66] utilized electroless plating to grow Au NPs and fill gaps between seeded Au NPs on a T-shaped DNA origami. They used a commercial Au plating solution with added Mg^2+^ to obtain continuous and conductive Au nanostructures. Uprety et al. [89] deposited Au NPs on individual DNA origami and then carried out electroless plating to create continuous Au NWs on one half of a bar-shaped DNA origami. They coated the gold NW surface with an octadecanethiol self-assembled monolayer as an organic mask. Next, they used Pd seeding and electroless Cu plating to create Cu NWs on the other half of the DNA origami to form Au-Cu bimetallic NWs. Stern et al. [98] fabricated 700 nm long NWs with a modified Au electroless plating solution having ascorbic acid to get a smooth coating with improved conductivity. These gold NWs displayed resistance under 3 kΩ and were stable for over one year, still showing conductivity after 16 months. However, if the gold-coating layer was <10 nm, only 20% of the wires were conductive along their full length. Almaky et al. [80] produced metal shell and polymer core NWs on DNA by decorating poly N-(3-pyrrol-1-yl-propyl)-2,2′-bipyridinium hexafluorophosphate coated DNA using electroless deposition of Cu or Pd. However, the scanning conductance microscopy measurements showed only the Cu ones had uniform phase shifts.

**Figure 4:**
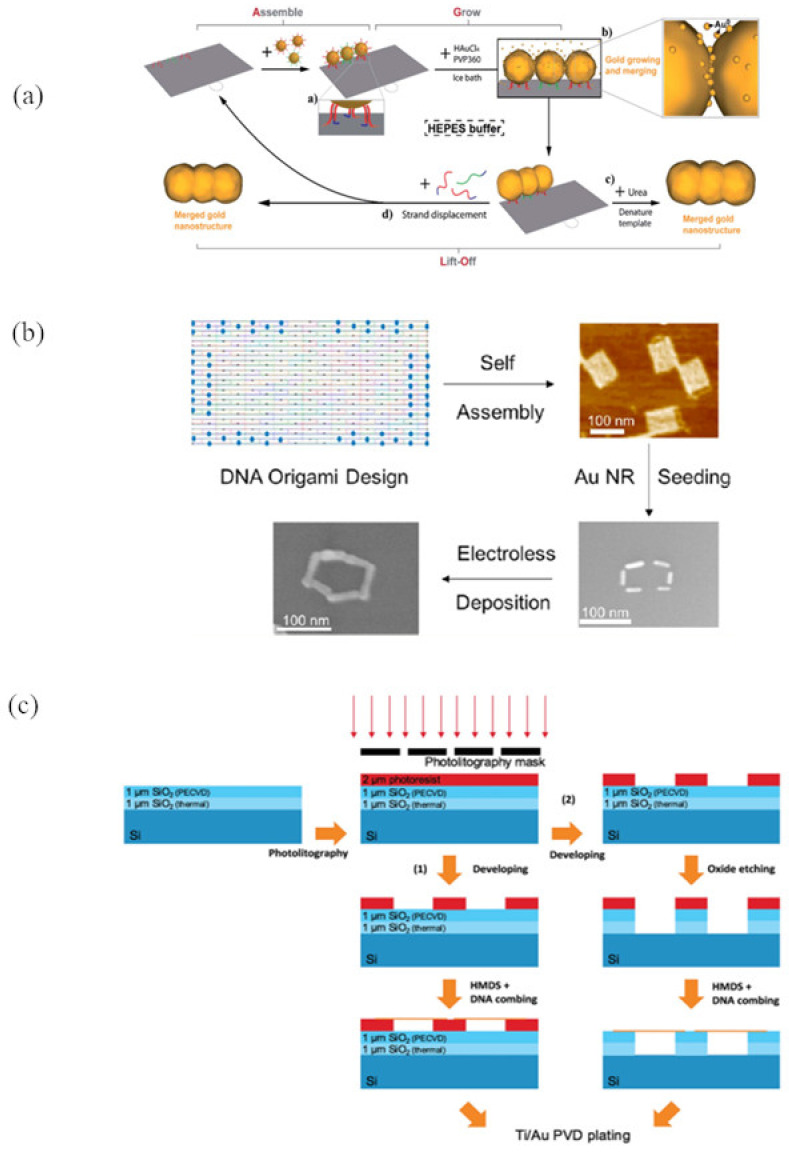
Plating technologies. (**a**) Schematic illustration of employing the assemble, grow, and lift-off strategy to construct a pre-designed gold nanostructure. Reprinted from Luo et al. [55] Copyright 2020 The Royal Society of Chemistry. (**b**) Formation of a continuous rectangular Au nanostructure by attaching Au NRs to DNA origami. Reprinted from Uprety et al. [91] Copyright 2017 American Chemical Society. (**c**) Process flow presenting electrode fabrication made by photoresist (1) and silicon dioxide (2), followed by suspending DNA between the electrodes, and metallization by vapor deposition. Reprinted from Brun et al. [35] Copyright 2017 Wiley-VCH Verlag.

Coupling of site-specific deposition and electroless plating is promising for assembly of designed metal nanostructures. Luo et al. [55] reported the creation of gold nanostructures by depositing Au NP seeds onto pre-designed locations on 2D DNA origami (Figure 4a). Continuous standalone gold nanostructures in custom shapes were generated by aggregating gold onto the surface of the Au NP seeds. These gold nanostructures can be lifted off and easily removed from the DNA template using a urea wash and ultrasonication. This strategy recycles the DNA origami template and reuses the excess Au NP seeds, which could reduce materials costs and save time. Future efforts should test whether this approach could be used for more complex 3D nanostructure construction.

Electroless plating can be used for growth of metal NRs in addition to metal NPs. Uprety et al. [90] utilized electroless plating to grow Au NRs, resulting in conductive nanowires with 13–29 nm widths and 8.9 × 10^−7^ Ω·m resistivity. In addition, Uprety et al. [91] and Aryal et al. [60] assembled different kinds of nanostructures such as plus, cross, and C shapes using Au NR seeds on DNA origami templates. They further utilized electroless plating to grow gold NRs, resulting in continuous rectangular, square, and T-shaped metal structures on tile DNA origami (Figure 4b). Moreover, Aryal et al. [60] utilized electroless plating of Au NRs to fill gaps of up to 10 nm between seeds to assemble conductive plus, cross, and C-shaped Au NWs on DNA origami tiles.

### 4.2. Galvanic Displacement

Galvanic displacement is a subcategory of electroless deposition, serving as the first step of electroless plating. Galvanic displacement can be used for batteries and for energy storage or conversion [5]. Liu et al. [99] utilized galvanic displacement on Ni and Cu NWs to form continuous Te NWs on λ-DNA. Acidic solution containing HTeO_2_^+^ caused galvanic displacement of the Ni and Cu, resulting in a Te coating. The synthesized NWs were influenced by the composition of the displaced metal. 

### 4.3. Photochemical

Photochemical deposition involves a redox reaction that uses UV light as the energy source. This technique is simple and inexpensive, so it can be used in large-scale processes such as solar cell fabrication. Because of nucleation effects, the reaction products tend to form NPs, which may be useful for processes such as gas sensor fabrication [100]. Hossen et al. [54] utilized photochemical deposition to coat Ag on the surface of triangular DNA origami. The DNA templates served as localized photosensitizers activated by UV light, and the Ag^+^ from the solution was reduced and grew on the template surface. This metallization process was self-limiting, as growth was terminated when all the surface area of the DNA template had been coated. 

### 4.4. Physical Vapor Deposition

Physical vapor deposition is a vacuum process for transferring materials from a solid material source to the gas phase in a vacuum chamber, then depositing them back as a solid on the substrate surface [101]. Brun et al. [35] used physical vapor deposition to deposit Ti and Au onto dsDNA to assemble NWs as shown in Figure 4c. The NWs were 60–80 nm thick and conductive; they were seeded on silicon, with potential for use in the microelectronics industry. However, the thickness of the Ti/Au coating was not uniform, ranging from 16–80 nm, which affected continuity. 

We summarize details of seeding and plating in Table 2, which includes methods of seeding and plating, dimensions and forms of seeds, geometry of templates, yield of fabrication, and potential applications of the resulting nanostructures.

## 5. Non-Metallic Conductors on DNA Nanostructures

### 5.1. Semiconductors

**Figure 5:**
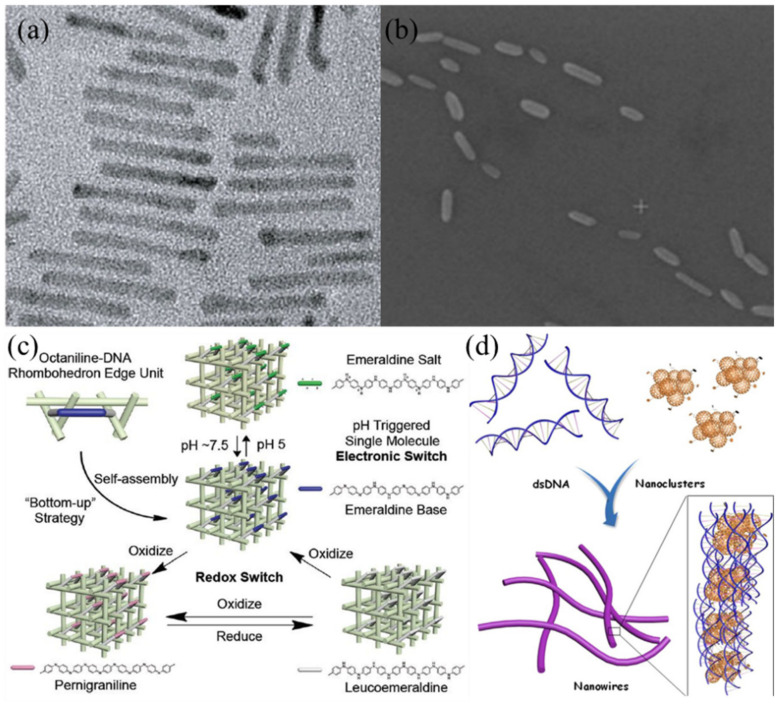
Semiconductors in DNA-based nanofabrication. (**a**) CdS NRs. Reprinted from Weichelt et al. [67] Copyright 2019 Wiley-VCH Verlag. (**b**) Te NRs. Reprinted with permission from Aryal et al. [56] Copyright 2020 Springer. (**c**) Octaniline organic semiconductor assembly with DNA. Reprinted from Wang et al. [104] Copyright 2017 Wiley-VCH Verlag. (**d**) Fullerene clusters associated with DNA strands. Reprinted from Vittala et al. [102] Copyright 2017 Wiley-VCH Verlag.

Incorporation of semiconductor materials along with metals on DNA platforms enables bottom-up fabrication of nanoelectronic building blocks. Weichelt et al. [67] demonstrated the assembly of oligonucleotide-functionalized semiconducting 5 nm × 45 nm CdS NRs in DNA origami molds preloaded with Au NPs. Figure 5a shows the semiconducting CdS NRs; electroless deposition of gold was used to bridge the end-to-end gaps between NPs and NRs, creating metal-semiconductor interfaces. This method is promising for generating electronic device structures, but electronic characterization of the CdS-Au interfaces is still needed. Aryal et al. [56] synthesized metal–semiconductor junctions by seeding Au and Te on DNA templates. DNA-coated Au NRs were seeded in specific locations on DNA origami via hybridization, and then CTAB-coated Te NRs were attached in the gaps via electrostatic interactions to create a Au–Te–Au pattern. The Te NRs were approximately 20 nm diameter and 70 nm long (Figure 5b), and were interfaced with Au NRs ~10 nm in diameter and 45 nm long. The methods used in this work offer new opportunities for future fabrication of DNA origami-templated transistors and logic gates. 

DNA origami has also been used as a template for nanopatterning of semiconductors. Choi et al. [103] fabricated 2D transition metal dichalcogenide nanostructures by using DNA origami nanotubes as etch masks. DNA nanotubes were deposited onto 2D planes of MoS_2_, MoSe_2_, WS_2_, or WSe_2_, followed by metallization of the DNA nanotubes with Pd. Dry etching of the layers with XeF_2_ followed by dissolution of the Pd-DNA nanotubes in HCl exposed nanostructured semiconducting films. They observed photoluminescence and Raman signals from these nanostructured films, which could potentially be used in optoelectronics or optical devices.

Nurdillayeva et al. [105] applied inkjet printing to place CdS and λ-DNA onto surfaces; reaction between Cd(NO_3_)_2_ and Na_2_S solutions in the presence of λ-DNA yielded CdS NWs. This CdS/λ-DNA “ink” was printed in the form of droplets onto glass, creating a network of CdS NWs across Pt interdigitated electrodes. Inkjet printing could be a useful method to fabricate more complex DNA-templated semiconductor nanostructures in the future. 

Organic semiconducting materials are useful for generating field-effect transistors (FETs), light emitting diodes and sensors on DNA. Han et al. [106] fabricated organic FETs by incorporating a semiconducting polymer, poly[3-(potassium-7-hexanoate)-thiophene-2,5-diyl], into a DNA matrix. The strong π-π interactions between DNA and the semiconducting polymer during solution shearing resulted in the deposition of highly ordered semiconductor/DNA nanofibrillar structures. Moreover, DNA with Cu^2+^ doping increased the mobility of the semiconducting polymer to as high as 0.22 cm^2^V^−1^s^−1^. Zessin et al. [96] demonstrated site-specific positioning of BCPs comprised of polythiophene (π-conjugated p-type semiconducting polymer) and oligonucleotides on DNA origami on surfaces. A complementary linker in the DNA origami strands facilitated the binding of the DNA component of the BCP. Wang et al. [104] reported the self-assembly of an octameric aniline molecule, octaniline, an organic semiconductor, onto 3D DNA arrays (Figure 5c). To build these 3D octaniline-DNA crystals, the group first synthesized an octaniline-DNA conjugate, which was soluble in water. By exploiting the distinctive base sequence of each DNA sticky end, a 3D crystal of semiconducting octaniline was created. Although the authors did not perform electronic characterization of the crystals, this method could potentially be used to create complex electronic systems. Wang et al. [107] further constructed a DNA origami enabled electro-optical modulator using two organic semiconductors, octaniline and poly(phenylenevinylene) heptamer. Organic semiconductor–DNA conjugates were assembled onto DNA origami templates to form ‘X’ structures that manipulated energy transfer between the two regions. Electrical behavior of the structures was not studied, but the construction approach could be beneficial for fabricating organic molecular electronics.

Arranging semiconducting NWs on DNA and converting DNA structures into semiconducting nanomaterials are important for designing and making DNA-templated semiconductor devices. Vittala et al. [102] constructed semiconducting NWs by assembling fullerene clusters with short DNA strands. Figure 5d depicts 3–5 nm diameter nanoclusters of an aniline-associated fullerene derivative non-covalently interacting with DNA through groove binding and intercalation, generating micron-length NWs with an average width of 3 nm. Conductive AFM revealed nonlinear I-V curves for the NWs, indicating semiconducting properties, which could be useful for generating DNA electronics. Al-Mahamad et al. [108] prepared a 1D coordination polymer that exploited self-assembly of Au^+^ and 6-thioguanosine. The molecular structure of the polymer was analogous to duplex DNA and possessed semiconducting properties, which were confirmed by non-linear I-V curves from electrical characterization.

### 5.2. Carbon Nanotubes

**Figure 6:**
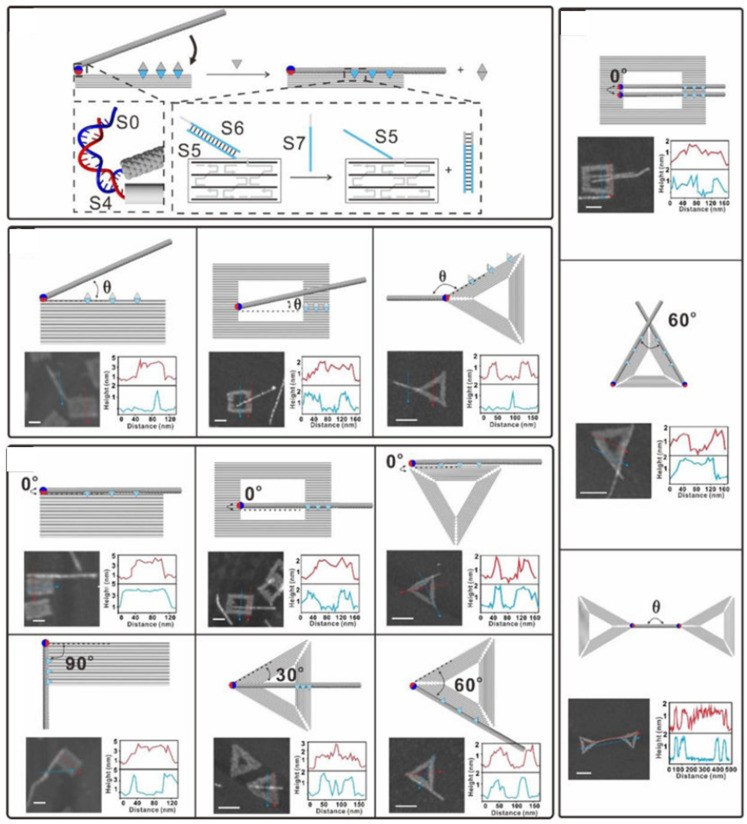
DNA-related assembly of CNTs. Placement of DNA end-functionalized CNTs onto DNA origami templates with different angles. Reprinted from Pei et al. [109] Copyright 2019 American Chemical Society.

**Figure 7:**
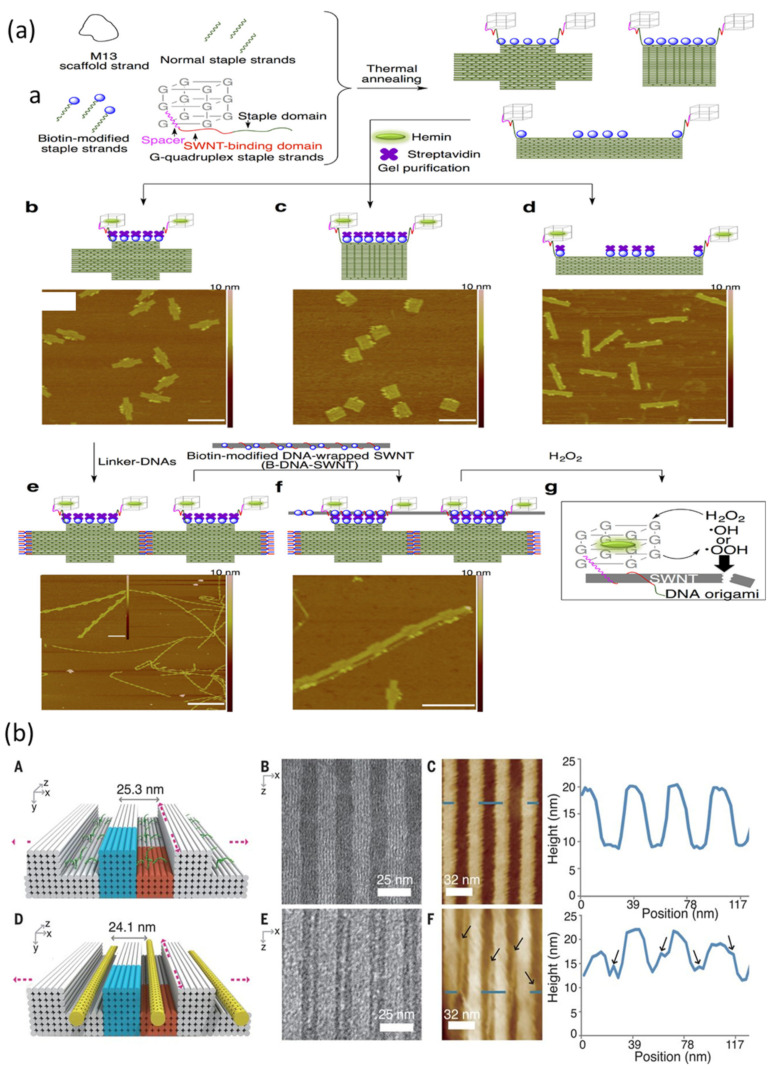
DNA-related assembly of CNTs. (**a**) DNA origami platforms for cutting CNTs with designated lengths. Reprinted from Atsumi et al. [111] Copyright 2018 American Chemical Society. (**b**) Assembling CNT arrays with controlled inter-CNT pitch. Reprinted from Sun et al. [70] Copyright 2020 American Association for the Advancement of Science.

Integration of CNTs into complex top down electronic structures requires precise CNT alignment to specific locations on a substrate. Pei et al. [109] showed CNTs attached onto triangular and rectangular DNA origami (Figure 6) with 1–3% yield, which could be increased by using smaller DNA components with larger limiting concentrations. Zhang et al. [110] reported CNT alignment at prescribed sites on DNA origami using DNA-linked Au NPs. They found that cooperative DNA hybridization occurred at the interface of DNA-coated Au NPs and CNTs, leading to an approximate five-fold improvement of the positioning efficiency. By combining this cooperative DNA hybridization with the intrinsic placement of DNA origami, CNTs can be aligned in parallel with an angular variation less than 10 degrees. Moreover, the study demonstrated that the parallel alignment of CNTs prevents incorrect logic functionality originating from stray conducting paths formed by misaligned CNTs. Controlling the length of CNTs is desirable for controlling and exploiting quantum effects in CNT devices. Atsumi et al. [111] developed an approach, shown in Figure 7a, to cut CNTs to predetermined lengths, facilitated by DNA origami-wrapped CNTs with improved dispersibility in water. G-quadruplex DNA, which is a natural DNA nanoarchitecture, forms a complex with hemin and activates hydrogen peroxide for CNT cutting.

CNTs and DNA can interact to form complex structural patterns. Zhang et al. [112] demonstrated sequence-specific DNA condensation onto CNTs, creating different patterns, heights, and distances. They further showed that these CNTs with tubular nucleic acid structures could be applied in information storage by attaching Au NPs with spacing controlled by the DNA condensation pattern. They were able to exploit the different structures for 2D encoding, with the information read using AFM imaging. This DNA and Au NP mediated method has potential for fabricating CNT arrays for nanoelectronics. Assembly of CNTs into densely aligned and evenly spaced arrays has attracted significant interest, as they could be integrated into light-emitting diodes, nanoelectromechanical oscillators, photodetectors, or chemical sensors. Sun et al. [70] demonstrated the use of DNA brick crystal nano trenches to align DNA-coated CNTs, as shown in Figure 7b. This method helped to build parallel CNT arrays with a consistent pitch as small as 10 nm.

CNTs are excellent candidates for use as transducers in electrochemical sensors because of their combination of high electron mobility and electron localization on the CNT surface where they are strongly perturbed by their environment. Mirzapoor et al. [113] synthesized branched CNT structures using two ssDNA sequences attached to the ends of CNTs via a linker DNA. They used this approach for label-free conductivity detection to identify mismatches in hybridized DNA. CNTs functionalized with ssDNA have also attracted attention because of their high dispersion efficiency. Furthermore, CNTs are useful in fabricating FETs. Zhao et al. [114] utilized DNA-templated CNTs to enhance transport by tenfold compared to other FETs, by using spatially hindered integration of nanotube electronics. By comparing the DNA-containing and DNA-free FETs, they found that a high concentration of ssDNA and metal ions within multichannel FETs could decrease electrical conduction.

## 6. Characterization

An important aspect of materials work is characterization to determine whether the methods utilized yielded the expected structure with the desired functional properties. We divided characterization into two categories: structural and electrical. Most of the research cited so far performed some type of structural characterization, to confirm either the end result of the work or to verify the outcome of an intermediate step. For some of the cited works, the future goal is the use of DNA-based nanodevices in some form of nanoelectronics, which further requires an understanding of nanostructure electrical properties. Both structural and electrical characterization methods help in further advancing our understanding of DNA-templated electronic nanomaterials.

### 6.1. Structural Characterization

A common structural characterization technique for working with DNA nanostructures is atomic force microscopy, which uses a microcantilever raster-scanned across a surface to yield a nanoscale topographical map of a sample with sub-nanometer height resolution [115]. One challenge with AFM is its reduced accuracy in measuring lateral dimensions, due to tip-induced image broadening.

Another common method of DNA nanostructure characterization is scanning electron microscopy, particularly when inorganic materials have been deposited on the DNA. One advantage that SEM has over AFM is that the lateral resolution is much better, approaching a few nanometers. A common challenge with SEM imaging of DNA structures is the need for the imaged structures to be conductive for high-resolution imaging [116], and neither the DNA nor the substrate it is on are typically conductive. This challenge can be readily overcome by coating a sample with metal prior to imaging, or using, for example, only a thin oxide layer on a silicon substrate. Contrast can also be a significant challenge in imaging bare DNA as the relatively low Z elements in DNA weakly interact with the electron beam. Inorganics attached to the DNA are generally much easier to image in SEM.

Although not as common as AFM or SEM, transmission electron microscopy can also be useful for high-resolution DNA nanostructure imaging. TEM provides sub-nanometer resolution; an electron beam is passed through the sample, and the collected electrons form an image. A major challenge associated with TEM is the need for an “electron-transparent” substrate, through which electrons can pass freely without absorption or scattering, something not essential for either SEM or AFM. As an example of the power of this method, Yang et al. [117] used TEM to image the positions of DNA origami tiles with AuNPs to determine the state of AND and OR logic gates.

### 6.2. Electrical Characterization

**Figure 8:**
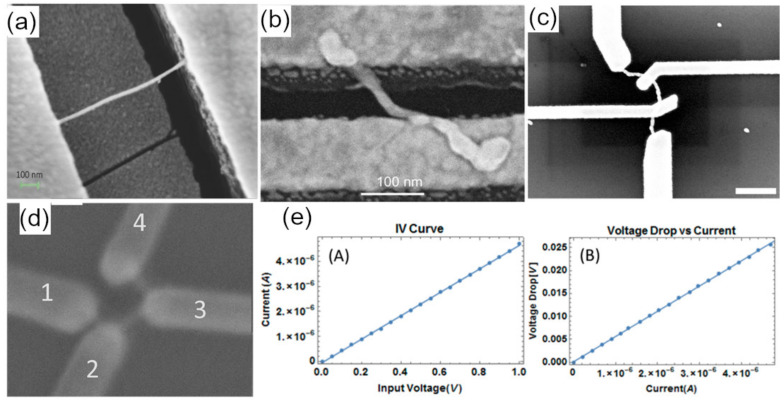
Electrical characterization of DNA-templated nanomaterials. (**a**) Two-terminal connection with patterns made via photolithography. Reprinted from Brun et al. [35]. Copyright 2017 Wiley-VCH Verlag. (**b**) Two-terminal connection patterned via EBL. Reprinted from Zhan et al. [63]. Copyright 2017 American Chemical Society. (**c**) Four-terminal connections patterned via EBL. Reprinted from Bayrak et al. [68]. Copyright 2018 American Chemical Society. (**d**) Four-terminal connections patterned via EBID. Reprinted with permission from Aryal et al. [60]. Copyright 2018 American Chemical Society. (**e**) I–V curves (left) for two-terminal measurement and (right) based on the voltage drop determined in four-terminal measurements. Reprinted with permission from Aryal et al. [60]. Copyright 2018 American Chemical Society.

Choosing how to obtain electrical characteristics is often dictated by the size and resistance of the conductive nanostructures. Prior to transport measurements, nanoscale terminal contacts are deposited onto the conducting nanostructure and the insulating substrate. A probe station can then be used to contact these deposited electrodes during the transport measurements. Patterning of the terminal contacts can be performed using several methods including photolithography (Figure 8a), electron-beam lithography (EBL) (Figure 8b,c), or electron-beam induced deposition (EBID) (Figure 8d). Conductance values can be obtained from I-V curves in both two-terminal measurements (Figure 8e, left) and four-terminal measurements (Figure 8e, right). In other instances, the goal is to determine if an electrically conductive structure (i.e., with continuity) has been made rather than measure an exact conductance value. For this type of test, characterization can be performed via less invasive methods, which include conductive AFM, electrochemical impedance spectroscopy, and scanning tunneling microscopy (STM).

### 6.3. Photolithography

Photolithography is ubiquitous in semiconductor manufacturing and is performed in a clean room. A pattern is formed by first spin coating a photosensitive polymer (photoresist) on the substrate containing the nanostructure, then exposing the resist with patterned UV light. Following UV exposure, the resist is immersed in a liquid developer that removes either the exposed (for positive resists) or unexposed (for negative resists) material. Photolithography resolution can go below 100 nm in large-scale industrial settings, but in most research environments resolution down to 1 µm or slightly smaller is achievable. Following lithography, two- or four-terminal contacts can be formed by deposition and lift-off of metal (commonly Au) to create a patterned thin film.

While not common for patterning contacts to many smaller DNA nanostructures, photolithography can be useful for contacting to NWs with lengths of 1 µm or more. Brun et al. [35] suspended DNA across photolithographically patterned interdigitated electrodes with gaps that ranged from 800–2000 nm as shown in Figure 8a. Once metallized, these NWs were found to be conductive, with resistances of 7–44 Ω.

### 6.4. Electron Beam Lithography

EBL can pattern a polymer electron beam resist with sub 50 nm resolution and is widely available to researchers with an SEM or a dedicated EBL instrument. After a thin polymer electron beam resist is spun onto the substrate, an electron beam is scanned across the substrate in a computer-designed pattern; locations where the beam hits the resist are exposed and then subsequently developed. As in photolithography, a positive or negative image in the resist allows for subsequent thin-film deposition and lift-off to create conductive contacts to the nanostructure.

One of the challenges of using EBL to contact bottom-up assembled nanostructures is alignment of the terminal contacts to the nanostructure. This is made worse by the fact that the process of imaging to locate and determine the orientation of a nanostructure exposes the resist in an undesired pattern. To address this matter, one can image the sample before resist deposition using pre-patterned alignment marks to reference the nanostructure location. Alternatively, a matrix of patterned metal can be created “blindly”, with the expectation that some nanostructures will make electrical contact by random placement. The matrix method works when there is a controlled density resulting in a significant fraction of electrodes with one and only one nanostructure attached for testing. Uprety et al. [90] used EBL to pattern interdigitated electrodes that had a small gap which NWs could randomly bridge. They identified the presence and number of gold NWs bridging between electrode sets and performed two-terminal electrical characterization on them. They found the resistivities of these 13–29 nm diameter nanowires to be as low as 8.9 × 10^−7^ Ω m.

As a precursor step to the formation of Te and Bi_2_Te_3,_ Liu et al. [99] made Ni NWs on DNA templates. To verify the continuity of the Ni NWs, they performed two-terminal electrical characterization with the contacts patterned using EBL. The contacts were closely spaced electrodes wherein the nickel NWs bridged the gap, and resistivities were measured to be between 1.0 × 10^−5^ and 4.8 × 10^−4^ Ω m when a single Ni NW spanned the gap.

In an effort to understand the transport mechanisms in NWs formed by the attachment of Au NPs on DNA, Teschome et al. [64] studied the temperature dependence of resistance. Using EBL, gold pads were used to contact a NW in a two-terminal configuration. Electrical characterization of the I-V relationship was performed in vacuum at temperatures from 4.2 K to room temperature. At room temperature, two NWs had resistances of 116 MΩ and 2.8 GΩ, which were unusually high compared to those of seeded and plated nanowires from other studies.

Although alignment with EBL is often a challenge, Tapio and Toppari [118] used gold electrodes patterned via EBL to perform dielectrophoresis and trap individual DNA origami, which then served as a template for seeding Au NPs that were grown into NWs. They observed non-linear I-V curves, attributed to the Coulomb blockade phenomenon.

To get accurate I-V data on low-resistance NWs, four-terminal measurements are required to eliminate lead and contact resistance, wherein current is applied using the two outer terminals and voltage is measured across the two inner terminals. This results in minimal lead or contact resistance contributions, as long as the lead and contact resistance is significantly smaller than the input impedance of the voltage measurement device. For nanostructures four-terminal measurements require high precision in patterning. Although EBL can pattern lines below 50 nm in width, alignment is very challenging on these scales. To determine the conductivity of gold NWs formed in DNA origami molds, Bayrak et al. [68] used highly aligned EBL (Figure 8c) in a four-terminal configuration, and measured resistances as low as 90 Ω for NWs that were 800 nm long.

### 6.5. Electron Beam Induced Deposition

Electron beam induced deposition is a direct patterning technique performed in a scanning electron microscope. The electron beam breaks apart an organometallic precursor gas, depositing metal atoms [119]. EBID is valuable because it allows for in-situ imaging and writing to a nanostructure, without requiring alignment marks. Like EBL, the feature size in EBID is limited by the size of the electron beam (broadened by scattered electrons), which leads to a resolution of about 25 nm. A downside with EBID is that the deposited metal is contaminated with carbon, increasing the resistance; this problem can be addressed with additional SEM purification steps. Aryal et al. [60] reported using EBID of platinum to make four-terminal connections to “C”-shaped, Au-plated nanostructures formed on a 70 × 90 nm^2^ DNA origami, and measured resistivities as low as 4 × 10^−5^ Ωm (Figure 8d,e). Aryal et al. [56] later used EBID of platinum to create electrodes in a two-terminal configuration to measure electrical properties of DNA-templated metal-semiconductor junctions. They verified electrical continuity between Au and Te NRs and measured I-V curves that showed Schottky-like behavior.

Westover et al. [120] evaluated temperature effects on DNA-templated Au NWs encased in EBL resist, as a way to possibly improve conductivity through polymer constrained annealing. Using four-terminal contacts made by EBID of tungsten, they found that at moderate temperatures (<200 °C), Au NWs exhibited morphological changes that were influenced by the presence of a polymer coating. The Au NWs had resistances as low as 153 Ω, and the resistance underwent large changes upon annealing.

### 6.6. Conductive AFM and STM

Conductive AFM or STM can measure the current flow from the conductive probe tip to the sample to measure some electrical properties of a sample. In conductive AFM, force interactions are used to take a topographical image of the sample surface while also measuring current from the tip to the sample. In STM, tunneling conductance from the tip to the surface is used to determine surface topography. Both measure vertical conductance of structures on the surface but do not measure lateral conductivity. For example, resistance through a nanoparticle to the substrate can be measured but the electrical continuity between touching or near touching nanoparticles cannot be determined. This can be remedied by performing conducting AFM on a substrate with patterned electrodes that contact only part of the nanostructure. Mohamed et al. [83] studied DNA-templated Rh NWs by first doing scanning conductance microscopy on the NWs to verify conductivity and then conductive AFM at different positions to get resistance values, from which they calculated the resistivity of the NWs to be 41–65 Ω cm. Yamada et al. [84] used an STM-break junction method to find conductance values possibly corresponding to three different conformations in metallo-DNA duplexes. A hypothesis as to specific structural conformations could have been a valuable further contribution to the paper.

### 6.7. Electrochemical Impedance Spectroscopy

Electrochemical impedance spectroscopy (EIS) applies an alternating current potential whose frequency is varied over a specified range, and measures the current response, phase shift and amplitude for the electrochemical system of interest. By fitting the acquired data to a lumped element model, best-fit model capacitance and resistance values for the sample can be determined. In a study of a way to make polyaniline on a DNA template, Giacobbe et al. [121] used EIS to determine the solution conductivity. They were able to make conductive polyaniline in an environmentally friendly way on a DNA template using laccase as a catalyst. They found an unexpected result: the DNA dopes the polyaniline such that the measured conductivity was much lower than expected from normal polyaniline.

A summary of the electrical characterization results presented here is found in Table 3. Each method for electrical characterization has pros and cons, such that a key consideration in deciding on an approach is to know what information is needed. For example, verifying electrical continuity can be achieved without collecting quantitative resistivity data to compare the resistivity to the bulk material. For NWs, resistivity is especially useful information in comparing NWs of different size. In future studies we encourage authors to report resistivity (and/or wire dimensions) where feasible.

## 7. Conclusions and Outlook

Recent achievements in DNA nanotechnology involve designing strategies and creating complex and functional DNA-based architectures in association with other inorganic or organic nanomaterials with optimized fabrication techniques. Similar to other DNA technology application fields such as medicine, imaging, and sensors, bottom-up fabrication of DNA-assembled conductive structures along with their electrical characterization is growing rapidly. We have reviewed fundamental steps associated with bottom-up fabrication of DNA-templated electronic nanomaterials and different characterization approaches to study a wide range of techniques employed for generating continuous structures on diverse DNA templates, and the resulting nanomaterial electrical properties. The programmable, precise, and predictable binding specificity of DNA molecules makes revolutionary changes possible utilizing DNA-assisted nanomaterials. DNA architectures that offer directed positioning of heterogeneous nanomaterials with customized orientations are ideal platforms to perform nanofabrication via self-assembly. Such sophisticated and ordered DNA-based nanostructures have displayed preliminary but promising applications in numerous research fields. For example, multiple material attachment on DNA structures provides unprecedented opportunities to fabricate functional electronic structures or devices such as nanowires, metal-semiconductor structures, transistors, logic gates, and ultimately integrated circuits in the future. Integration of photonic and electronic materials on DNA structures offers opportunities to explore physics and develop sensing, plasmonic, or surface-enhanced Raman scattering sensors. In addition, nanomedicine and therapeutics can take advantage of DNA nanostructures by utilizing them in photothermal therapy against cancer cells. Different types of DNA templates can also serve as masks to create nanopatterns on substrates for lithography, or further act as a nano-breadboard in forming functional components or devices.

Current progress in placing nanomaterials onto DNA templates demands innovative fabrication methodologies. Although we lack the technology to fully replicate many natural phenomena, we can use these phenomena to guide the development of new techniques and skills to improve nanofabrication. Advancements associated with DNA-templated nanomaterials will keep growing, providing increased knowledge and better procedures for their creation and characterization. To develop industry-friendly methods, we need further scientific improvements in: (a) the design and self-assembly of DNA scaffolds into complex structures, (b) mechanisms of precise and controlled placement of heterogeneous nanomaterials onto DNA platforms, (c) the creation of composite and conductive networks such as integrated circuits, and (d) effective characterization techniques to ensure next-generation nanoelectronics can be fabricated using DNA. In an interdisciplinary field, these improvements require collective efforts from many scientific specialties to mitigate challenges related to alignment, precision, and yields. DNA nanotechnology should be able to move the beyond present issues described herein and powerfully influence the nanoelectronic domain in the future.

## Figures and Tables

**Table 1:** Summary of DNA templates with dimensions and shapes, along with substrates and attached nanomaterials.

DNA Template	Dimension	Shape	Substrate	Attached Material(s)	Reference
**Single-Stranded**	2D	Linear	NA	CNT	[53]
**Double-Stranded**	3D	Lattice	NA	Au	[54]
	2D	Linear Wire	SiO_2_	Al/Au	[35]
**DNA Origami**	2D	Sheet (Tile)	Mica/Silica	Au	[55]
	2D	Bar	SiO_2_	Au/Te	[56]
	2D	Rhombus	Mica	Au	[37]
	2D	Bowtie	SiO_2_	Au	[57]
	2D	Triangle	NA	Pd/Fe_2_O_3_	[58]
	2D	Bowtie	SiN/Al_2_O_3_	Au	[59]
	2D	Tile	SiO_2_	Au	[54]
	3D	Rhombohedral	NA	Au	[60]
	2D	Bowtie	Si/Glass	Au	[61]
	3D	Tripods	Si	Au	[62]
	2D	Bar	SiO_2_	Au	[63]
	3D	Tubular Bundle	NA	Au	[35]
	2D	Planet-Satellites	Solid	Ag-Au/Organic	[36]
	2D	Tubular Bundle	Si	Au	[39]
	2D	Triangle	Si	Au/Ag	[64]
	2D	Toroidal	Si	Au core/Ag shell	[32]
	2D	T Shape	SiO_2_	Au	[38]
	2D/3D	Tile/Bowtie/Tube	Si/Glass	Au	[65]
	3D	DNA Molds	Si	Au	[66]
	3D	DNA Molds	Si	Au	[67]
	3D	Long DNA Molds	Si	Au	[68]
	2D	Triangle/Rectangle	Mica	NA	[47]
	2D	Triangle	Si	Au Film	[49]
	2D	Hexagonal Lattices	Mica	Proteins	[69]
	2D	DNA Tube	PMMA/PLLA	CNT	[40]
	3D	DNA Brick	-	CNT	[70]

CNT: Carbon Nanotube; PMMA: poly(methyl methacrylate); PLLA: poly(L-lactic acid).

**Table 2:** Summary of seeding and plating techniques, including form and size of seeds, coating material, template geometry, potential applications, and structure yield.

Material	Form of Seed	Seed Size (nm)	Seed Coating	Seeding Technique	Template Geometry	Plating Technique	Potential Applications	Final Structure Yield	Ref.
**Au** **Te**	NP NR	45 ± 9, 9 ± 2 72 ± 15, 21 ± 3	DNA CTAB	site-specific	linear	electroless	nanoelectronics	>60%	[56]
**CdS** **Au**	NR NP	45 × 5 5	DNA	site-specific	linear	electroless	nanoelectronics	70 ± 7%	[67]
**Fullerene-aniline**	NP	3.2	Aniline	non-covalent interactions	linear	N/A	nanotechnology	N/A	[102]
**Au**	NP	12	DNA	site-specific	linear, triangle, Y and L-shape, square	electroless	nanoelectronics and nanoplasmonics	>80%	[55]
**Au**	Nano-prism	80	DNA	site-specific	bowtie	N/A	photonics	62%	[62]
**Au**	NP	5	DNA	site-specific	linear	electroless	nanoelectronics	N/A	[68]
**Au**	NP	17, 28, 42, 56	DNA	site-specific	linear	N/A	optoelectronics/nanomedicine	17%	[36]
**Au**	NP	5, 10	DNA	site-specific	tetragonal, hexagonal	N/A	plasmonics	81–94%	[65]
**Au**	NP	10	DNA	site-specific	cluster and honeycomb lattices	N/A	biomedical	N/A	[92]
**Au**	NR	6 × 25	CTAB	electrostatic	linear	electroless	nanoelectronics	57%	[63]
**Rh**	ion	N/A	N/A	electrostatic DNA/Rh ions	linear	electrochemical	nanowires	N/A	[83]

**Table 3:** Summary of electrical characterization results.

Nanowire Material	Method of Contact	Nanowire Size	Resistance (R) or Resistivity (ρ)	Ref.
**Au**	EBL	13–29 nm × 400 nm	ρ = 8.9 × 10^−7^ Ω m	[90]
**Ni**	EBL	100 nm × 10 µm	ρ = 1.0 × 10^−5^ to 4.8 × 10^−4^ Ω m	[99]
**Au**	EBL	30 nm × 412 nm	R = 120 MΩ to 2.8 GΩ	[64]
**Au**	EBL	10–15 nm	Conductive	[118]
**Au**	EBL	40 nm × 800 nm	R = 90 Ω	[68]
**Au**	EBID	10 nm × 130 nm	ρ = 4.24 × 10^−5^ Ω m	[60]
**Doped film**	Direct to silver pad	Thin film	R = 2–14 MΩ	[82]
**Au/Te**	EBID	17 nm × 400 nm	Continuous/semiconducting	[56]
**Au**	EBID	10–20 nm × 400 nm	R = 150 Ω	[120]
**Rh**	Conductive AFM	<10 nm diameter	ρ = 41–65 Ω cm	[68]
**PANI**	EIS	NA	R = 3.4–3.9 kΩ	[121]
**Au-thioguanosine**	Platinum electrodes	2 nm	Conductive after doping	[108]
**Au/Ti**	Photolithography	60–80 nm × 0.8–2 µm	R = 7.7–43 Ω	[35]

## Abbreviations

dsDNA	double-stranded DNA
ssDNA	single-stranded DNA
NW	nanowire
CNT	carbon nanotube
2D	two-dimensional
3D	three-dimensional
λ	lambda
AFM	atomic force microscopy
SEM	scanning electron microscopy
TEM	transmission electron microscopy
STM	scanning tunneling microscopy
EIS	electrochemical impedance spectroscopy
NP	nanoparticle
NR	nanorod
e-beam	electron beam
BCP	block co-polymer
CTAB	cetyltrimethylammonium bromide
FET	field-effect transistor
EBL	electron-beam lithography
EBID	electron-beam induced deposition
I-V	current-voltage
